# Can dietary intake protect against low-grade inflammation in children and adolescents?

**DOI:** 10.1016/j.bbih.2021.100369

**Published:** 2021-10-28

**Authors:** Melissa Bujtor

**Affiliations:** Institute of Psychiatry, Psychology & Neuroscience Division of Psychological Medicine Kings College London and Institute for Physical Activity and Nutrition Research, School of Exercise and Nutrition Sciences, Deakin University, Melbourne, Victoria, Australia

**Keywords:** Dietary intake, Dietary pattern, Macronutrients, Biomarkers, Inflammation, CRP, Cytokine, Interleukin, Children, Adolescent

## Abstract

In children and adolescents, chronic low-grade inflammation has been implicated in the pathogenesis of co- and multi-morbid conditions to mental health disorders. Diet quality is a potential mechanism of action that can exacerbate or ameliorate low-grade inflammation; however, the exact way dietary intake can regulate the immune response in children and adolescents is still to be fully understood. In this review, I discuss the current observational and interventional evidence that supports a potential therapeutic role for dietary intake in the amelioration of low-grade inflammation and highlight the need to develop a better understanding of the biological mechanisms underlying and attenuating the associations between dietary intake and low-grade inflammation in children and adolescents.

## 1 Introduction

Inflammation is a physiological response to cellular and tissue damage. It is designed to protect the host from bacteria, viruses and infections by eliminating pathogens, promoting cellular repair and restoring homeostatic conditions (Calder et al., 2011). However, a prolonged inflammatory state through chronic low-grade inflammation has deleterious effects, including irreparable damage to tissues and organs, and increased risk of disease status (Minihane et al., 2015).

Low-grade inflammation, reflected in the overproduction of acute-phase proteins such as C-reactive protein (CRP), pro-inflammatory cytokines such as interleukin-6 (IL-6) and tumour necrosis factor-alpha (TFN-α) has been established as a risk factor for several neuropsychiatric disorders (Giacobbe et al., 2020; Mitchell and Goldstein, 2014), including major depressive disorder (Colasanto, 2020), depression (Kiecolt-Glaser et al., 2015; Zunszain et al., 2012; Sawyer et al., 2019; Cattaneo et al., 2020; Pitharouli et al., 2021) and schizophrenia (Osimo et al., 2018). Moreover, low-grade inflammation in children and adolescents has been associated with the development of co- and multi-morbid conditions to mental health pathologies (Barbaresko et al., 2013; Furman et al., 2019; Stein et al., 2019; Bennett et al., 2018), including cardiovascular disease (Singh et al., 2015; Amaral et al., 2020), metabolic syndrome (Al-Hamad and Raman, 2017), type-II diabetes (Reinehr, 2019) and obesity (Stroescu et al., 2019), therefore making inflammation an important therapeutic target to study, especially for individuals suffering from those conditions.

The potential factors that promote low-grade chronic inflammation are diverse, and increasingly, attention has been given to diet quality as a potential mechanism of action that can exacerbate or ameliorate low-grade inflammation and subsequently influence mental health (Marx et al., 2020; Berk et al., 2013). Certainly, healthy dietary patterns of high quality, such as adherence to a Mediterranean Diet (Grosso et al., 2014), or eating foods such as vegetables and fruit (Calder et al., 2011), or macro/micronutrients, such as omega-3 polyunsaturated fatty acids (PUFAs) (Silveira et al., 2018) or vitamins C and E (Sun et al., 2011), respectively, have been shown to reduce systemic inflammation (Esposito et al., 2004; Giugliano et al., 2006). In contrast, the prevailing Western dietary pattern, which is high in refined grains, red meat, refined sugar and saturated fat, illicit elicits a pro-inflammatory response and increasing levels of circulating inflammatory biomarkers (Silveira et al., 2018).

Moreover, it is well established that a healthy diet in childhood and adolescence is crucial for optimal growth and development and for disease prevention (van der Velde et al., 2019). For example, higher vegetable intake in childhood has been associated with a lower risk of developing mental health pathologies later in life (Lassale et al., 2019), such as depression. In addition, a healthy diet can contribute to the prevention of cardio-metabolic multi-morbidities, often seen in adult patients with neuropsychiatric conditions. However, the efficacy of nutritional strategies for children and adolescents with mental health pathologies has been largely under-explored. While in observational and interventional studies, a higher quality, nutrient-rich diet, has been associated with a reduced risk of adverse mental health in both children (Kohlboeck et al., 2012) and adolescents (Jacka et al., 2010, 2011), to the best of the author's knowledge, there are presently no rigorous intervention studies explicitly designed to take a dietary approach to treatment in children and adolescent populations with existing adverse mental health. Further research studies are urgently needed.

Former literature reviews in children and adolescents have, for the most part, focused on various aspects of diet and various biomarkers that are not specifically related to the immune system function and response (Hilger-Kolb et al., 2017; Suhett et al., 2020; Rocha et al., 2017). In my recent review (Bujtor et al., 2021) I have presented several studies that bring together the current evidence base from observational and interventional studies investigating associations between dietary intake, through dietary patterns, food groups, macronutrients or micronutrients, and biological markers of low-grade inflammation, including CRP, IL-6 and TNF-α among others, in both children and adolescents. Taken together, these studies indicate a good quality diet, high in vegetable and fruit intake, whole grains, fibre and healthy fats ameliorates low-grade inflammation and therefore represents a promising therapeutic approach, as well as an important element for disease prevention in both children and adolescents. However, the mechanisms by which dietary patterns affect the inflammatory process are largely underexplored. In this review, I discuss the current evidence that supports a potential therapeutic role for dietary intake in the amelioration of low-grade inflammation and highlight the need to develop a better understanding of the biological mechanisms underlying and attenuating the associations between dietary intake and low-grade inflammation in children and adolescents.

## 2 Dietary intake and inflammation

### 2.1 Dietary patterns and indices

It is important to examine diet–disease relationships through dietary patterns, which are based on a countries dietary guidelines (Barbaresko et al., 2013), as foods are typically eaten in combination, and nutrients have both synergistic and antagonistic biochemical interactions (Tapsell et al., 2016). Dietary patterns capture the whole diet, including the combination of foods and nutrients (Moeller et al., 2007). The Mediterranean dietary pattern, examined in ten studies in this review (Del Mar Bibiloni et al., 2013; Liese et al., 2018; Agostinis-Sobrinho et al., 2019; Arouca et al., 2018; Carvalho et al., 2018; Douros et al., 2019; Karampola et al., 2019; Lazarou et al., 2010; Sureda et al., 2018; Çağiran Yilmaz et al., 2019) is characterized by a high intake of vegetables, fruit, whole grains, legumes, nuts, fish and low-fat dairy, alongside moderate to low consumption of meat and healthy fats (Dai et al., 2008). Results showed adequate adherence was associated with lower levels of CRP (Agostinis-Sobrinho et al., 2019; Sureda et al., 2018), IL-6, and TNF-α (Carvalho et al., 2018), and soluble vascular cell adhesion molecule-1 (sVCAM-1) (Arouca et al., 2018) in healthy males and females, and IL-17 (Douros et al., 2019) in both males and females with asthma. Whereas, the majority of studies conducted in cohorts with underlying pathologies, including type-1 diabetes (Liese et al., 2018), and obesity (Karampola et al., 2019; Lazarou et al., 2010; Çağiran Yilmaz et al., 2019), and one study in a healthy cohort (Del Mar Bibiloni et al., 2013) found no associations with inflammatory biomarkers. In interventional studies examining adherence to a low glycaemic index diet pattern (Rouhani et al., 2016) or to a hypocaloric high glycaemic index diet pattern (Rouhani et al., 2016; Iannuzzi et al., 2009; Parillo et al., 2012) adequate adherence was associated with lower levels of CRP, both in males and females with obesity, while another study found no association (Damsgaard et al., 2013). As well as in one intervention study in females with metabolic syndrome which examined the DASH diet and found adherence associated with lower levels of CRP, in female patients (Saneei et al., 2014), while a second observational study found no association (Liese et al., 2018) in neither males or females with type-1 diabetes. Taken together these results indicate that adequate adherence to healthful dietary patterns is associated with decreased levels of biomarkers, including CRP, IL-6 and TNF-α.

**Fig. 1:**
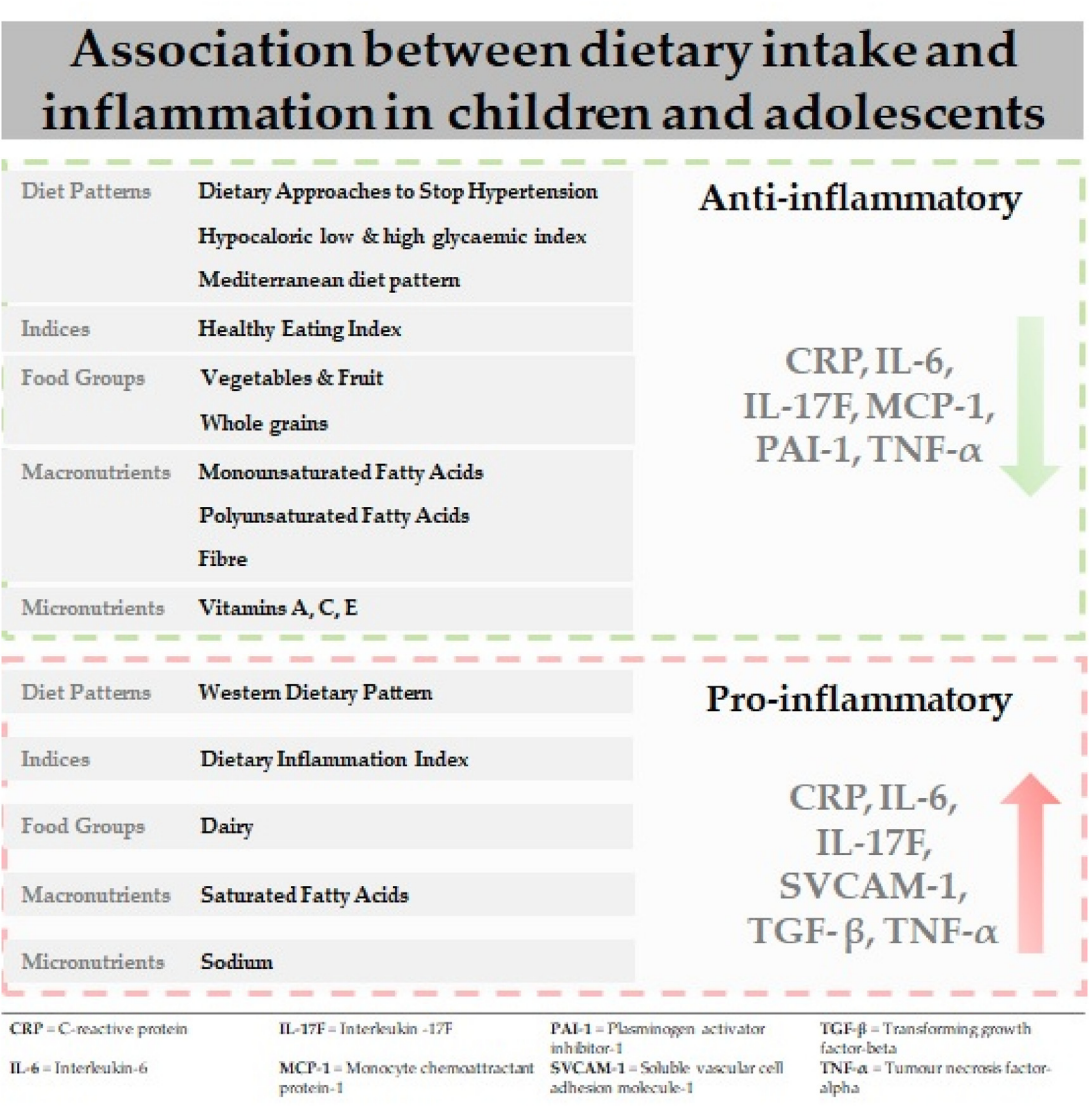
Association between dietary intake and biomarkers of inflammation in children and adolescents (Bujtor et al., 2021).

Dietary indices are nutritionally derived indices based on levels of consumption of nutrients or food groups (Bowyer et al., 2018). Of the five observational studies that examined healthful dietary indices, three observational studies focused on the Healthy Eating Index (HEI), a tool that assesses adherence to the Dietary Guidelines of Americans in any given year (Liese et al., 2018; Navarro et al., 2017; Sanjeevi et al., 2018). The first study found that a higher score in the HEI (healthy diet) was associated with lower levels of CRP in females, but not males from a healthy cohort (Navarro et al., 2017). In contrast, two studies found that moderate HEI scores (moderately healthy diet) were not associated with CRP or IL-6 in males or females in cohorts of patients with type-1 diabetes (Liese et al., 2018; Sanjeevi et al., 2018). The remaining two observational studies (Chan She Ping-Delfos et al., 2015; Vyncke et al., 2013) examined the Diet Quality Index (DQI), a composite individual-level diet quality indicator that enables cross-cultural diet quality comparisons. However, neither of the studies found an association between the DQI and CRP (Chan She Ping-Delfos et al., 2015; Vyncke et al., 2013) or IL-1, IL-6, IFN-y and TNF-α (Vyncke et al., 2013). This suggests a lack of sensitivity associated with the DQI as a measure of inflammatory status (Fig. 1).

In contrast to healthful dietary patterns, three studies examined the Western dietary pattern (Del Mar Bibiloni et al., 2013; Oddy et al., 2018; Khayyatzadeh et al., 2018). This pattern is characterised by high amounts of refined grains, red meat, high-fat dairy, ultra-processed food intake and trans fatty acids while being low in omega-3 PUFAs (Li et al., 2020; Okręglicka, 2015). Results showed positive associations with pro-inflammatory markers, specifically CRP and IL-6 in healthy males and females. The first study was longitudinal and found an association between WDP adherence at 14-years and higher levels of hs-CRP at 17-years both in healthy males and females (Oddy et al., 2018). The remaining two observational studies found an association between WDP adherence, and higher levels of CRP and IL-6 in this case in female cohorts of healthy participants (Del Mar Bibiloni et al., 2013; Khayyatzadeh et al., 2018). Similarly, across the six observational studies (Lazarou et al., 2010; Sen et al., 2018; Almeida-de-Souza et al., 2018; Coheley et al., 2019; Seremet Kurklu et al., 2020; Shivappa et al., 2017) that examined the Dietary Inflammatory Index (DII), a tool that assesses the inflammatory potential of a diet (Suhett et al., 2020), diets with high inflammatory potential (a higher score in the DII), inducing a higher inflammatory response were positively associated with pro-inflammatory biomarkers in males and females. Specifically, higher levels of hs-CRP, IL-6 and TNF-α in healthy males and females. Four of the studies found a positive association between adherence to a pro-inflammatory diet (indicated by a higher score in the DII) and IL-6 (Almeida-de-Souza et al., 2018; Seremet Kurklu et al., 2020), IL-1, IL-2, interferon gammon and sVCAM-1 in healthy males and females (Shivappa et al., 2017), but also a positive association between DII and CRP in males and females with obesity (Lazarou et al., 2010). However, two studies did not find any associations between the DII and CRP (Sen et al., 2018), or IL-6, MCP-1 or TNF-α (Coheley et al., 2019) in neither healthy males or females. This was likely due to study design and methodology. For example in one of the studies (Sen et al., 2018) exposure assessment (dietary intake) occurred approximately four years before outcome assessment (CRP) and given dietary patterns change rapidly in childhood (Demory-Luce et al., 2004) timing may have affected any potential associations. Furthermore, in this study, CRP data was only available in a small subset of children from the overall study population.

Overall, the results regarding dietary patterns and indices show adequate adherence to healthful dietary patterns, such as the DASH diet, low glycaemic index diets and the Mediterranean diet are associated with decreased levels of biomarkers, including CRP, IL-6 and TNF-α. While a Western dietary pattern and diets with high inflammatory potential as measured by the DII elicited a pro-inflammatory response increasing levels of the same biomarkers. Associations across the studies were attenuated by gender, as well as the presence of underlying pathologies, independent of dietary intake. However, the mechanisms by which dietary patterns affect the inflammatory process are largely underexplored (Marx et al., 2020). It has been previously hypothesised that the protective effect of healthful dietary patterns like the Mediterranean diet, and the pro-inflammatory response elicited by a Western dietary pattern may be derived from the properties of their constituents (Casas et al., 2014).

### 2.2 Food groups, macro- and micronutrients

The associations between the individual constituents of dietary patterns (food groups, macro- and micronutrients) and inflammatory markers are attenuated by gender, as well as the presence of underlying pathologies, independent of dietary intake. Concerning food groups, of the nine observational studies, examining vegetable and/or fruit intake (Arouca et al., 2018; Navarro et al., 2017; Sanjeevi et al., 2018; González-Gil et al., 2016; Qureshi et al., 2009; Hagin et al., 2017; Holt et al., 2009; Han et al., 2015; Cabral et al., 2018), two were previously discussed (Navarro et al., 2017; Sanjeevi et al., 2018). Notably, across the studies high dietary intake of vegetables and/or fruits was associated with lower levels of CRP (González-Gil et al., 2016; Qureshi et al., 2009; Hagin et al., 2017) and IL-6 (Holt et al., 2009; Cabral et al., 2018) (both in healthy males and females), TNF-α (Arouca et al., 2018) (only in healthy females), and IL-17F (Han et al., 2015) (both male and female patients with asthma). In contrast, one study found an association between intake and lower levels of hs-CRP in females, but not in males (Navarro et al., 2017), while one study did not find any associations (Sanjeevi et al., 2018). Whole and refined grain intake were examined in one intervention (Hajihashemi et al., 2014) and six observational (Arouca et al., 2018; González-Gil et al., 2016; Qureshi et al., 2009; Han et al., 2015; Cabral et al., 2018; Hur and Reicks, 2012) studies, five of which were previously discussed (Arouca et al., 2018; González-Gil et al., 2016; Qureshi et al., 2009; Han et al., 2015; Cabral et al., 2018). Overall, these studies found that wholegrain dietary intake was associated with lower levels of CRP in a female cohort with obesity (Hajihashemi et al., 2014) and in healthy males and females (Hur and Reicks, 2012) as well as IL-17F in males and females from cohorts with and without underlying pathologies (Han et al., 2015). In contrast, results on the association between refined grain intake and inflammatory markers remain inconclusive (Arouca et al., 2018; González-Gil et al., 2016; Qureshi et al., 2009; Cabral et al., 2018). Similarly, dairy was examined in seven observational studies (Arouca et al., 2018; Sanjeevi et al., 2018; González-Gil et al., 2016; Qureshi et al., 2009; Han et al., 2015; Cabral et al., 2018; Aslam et al., 2020), six of which were previously discussed (Arouca et al., 2018; Sanjeevi et al., 2018; González-Gil et al., 2016; Qureshi et al., 2009; Han et al., 2015; Cabral et al., 2018). While one study found an association between dairy intake and higher levels of IL-17F in males and females, from a cohort of patients with asthma (Han et al., 2015) and a second study found an association between dairy intake and higher levels of IL-6, Il-10 and TGFβ-1 in females, but not in males, and IL-1 in both males and females, and IL-5 in males but not in females in a healthy cohort (Arouca et al., 2018) the majority of studies did not find any associations with inflammatory biomarkers (Arouca et al., 2018; Sanjeevi et al., 2018; González-Gil et al., 2016; Qureshi et al., 2009; Han et al., 2015; Cabral et al., 2018).

Even more interesting is the fact that the majority of studies examining pro-inflammatory foods (Tabung et al., 2016; Kanauchi et al., 2019) including meat, seafood and eggs (Arouca et al., 2018; Sanjeevi et al., 2018; González-Gil et al., 2016; Qureshi et al., 2009; Han et al., 2015; Cabral et al., 2018; Aeberli et al., 2006), and added sugars (snacks—candy, jams, spreads, sugar-sweetened beverages, fruit juice) (Karampola et al., 2019; Sanjeevi et al., 2018; González-Gil et al., 2016; Hagin et al., 2017; Holt et al., 2009; Han et al., 2015; Cabral et al., 2018; Kosova et al., 2013; Jin et al., 2014) did not find an association between dietary intake and CRP, IL-6 and TNF-α in males and females from healthy cohorts. This is particularly interesting in the case of meat, which has previously demonstrated one of the most consistent epidemiological associations between diet and human disease risk (Alisson-Silva et al., 2016) and has been associated with increased levels of CRP in other studies (Montonen et al., 2013). The lack of associations between these constituents and inflammatory markers in the studies in this review may be due to the observational study design and the methodology employed. It is widely accepted that nutrition epidemiology studies are affected by reporting bias. Imprecision in the measurement of dietary intake is often observed, particularly in children and adolescents, which can cause the over- or under-estimation of the impact of exposure (Livingstone and Robson, 2000; Foster and Adamson, 2014; Magarey et al., 2011). Whereas biological, rather than self-reported, dietary measures are a more accurate and reliable way of investigating dietary intake as well as absorption (Potischman, 2003)and should be more often used in future research studies (Dragsted et al., 2018).

In terms of macronutrients, fat was examined in 13 observational studies (Del Mar Bibiloni et al., 2013; Arouca et al., 2018; Karampola et al., 2019; Navarro et al., 2017; Sanjeevi et al., 2018; Qureshi et al., 2009; Aeberli et al., 2006; Prihaningtyas et al., 2019; Thomas et al., 2008; Harris et al., 2019; Arya et al., 2006; Oldewage-Theron and Kruger, 2017; Au et al., 2012), 7 of which were previously discussed (Del Mar Bibiloni et al., 2013; Arouca et al., 2018; Karampola et al., 2019; Navarro et al., 2017; Sanjeevi et al., 2018; Qureshi et al., 2009; Aeberli et al., 2006). Overall, the studies found that saturated fatty acids intake was associated with higher levels of CRP in healthy females but not males (Navarro et al., 2017; Arya et al., 2006) as well as obese males and females (Aeberli et al., 2006) and healthy males but not females (Harris et al., 2019). One study found an inverse association between monounsaturated fatty acid (MUFA) and IL-6 as well as between omega-3 polyunsaturated fatty acid and plasminogen activator inhibitor-1 in healthy females (Del Mar Bibiloni et al., 2013). Another study found a positive association between MUFA : SFA ratio and IL-6 in males and females, TGFβ-1 in females but not in males, and sVCAM-1 in males but not in females, from a cohort of healthy participants (Arouca et al., 2018). The same study also found an inverse association between SFA and sVCAM-1 in females but not in males (Arouca et al., 2018). Lastly, seven studies found no associations between SFA and CRP (Sanjeevi et al., 2018; Thomas et al., 2008) and total fat and CRP (Karampola et al., 2019; Qureshi et al., 2009; Prihaningtyas et al., 2019; Oldewage-Theron and Kruger, 2017; Au et al., 2012) or IL-6 (Au et al., 2012). Fibre was examined in one intervention (Machado et al., 2015) and nine observational (Karampola et al., 2019; Navarro et al., 2017; Qureshi et al., 2009; Arya et al., 2006; Jaacks et al., 2014; Lin et al., 2015; Swann et al., 2020; Parikh et al., 2012; Miller et al., 2016) studies, three of which were discussed previously (Karampola et al., 2019; Navarro et al., 2017; Qureshi et al., 2009). Results show that seven studies did not find an association between fibre intake and CRP in males or females from healthy cohorts (Qureshi et al., 2009; Arya et al., 2006; Machado et al., 2015; Jaacks et al., 2014; Lin et al., 2015; Swann et al., 2020) and an obese cohort (Karampola et al., 2019).Whereas three studies found an association between fibre intake and lower levels of CRP (Navarro et al., 2017; Parikh et al., 2012), from cohorts of healthy participants and lower levels of plasminogen activator inhibitor-1, and monocyte chemoattractant protein-1 in overweight males and females (Miller et al., 2016). Lastly, a total of seven observational studies examined various micronutrients (Del Mar Bibiloni et al., 2013; Navarro et al., 2017; Holt et al., 2009; Oldewage-Theron and Kruger, 2017; Zhu et al., 2014; King et al., 2007; de Sousa et al., 2019), three of which were discussed previously (Navarro et al., 2017; Holt et al., 2009; Oldewage-Theron and Kruger, 2017). These studies found an association between dietary intakes of vitamins, specifically A, C and E, and lower levels of CRP and IL-6, while sodium was associated with higher levels of TNF-α, in males and females from a cohort of healthy participants (Zhu et al., 2014). Taken together these results indicate that high intakes of vegetables, fruit and whole grains resulted in lower levels of inflammatory biomarkers, such as CRP, IL-6 and TNF-α. The same was for healthy fats such as MUFA and PUFA and various micronutrients such and vitamins A, C and E, all of which are considered to have anti-inflammatory properties (Schultz et al., 2019). While higher intakes of saturated fatty acids and sodium elicited an inflammatory response.

Indeed, the Mediterranean diet is characterized by high intakes of anti-inflammatory food groups, macro- and micronutrients (Dai et al., 2008). The diet is rich in vegetables, fruits and whole grains and therefore also antioxidants, folate, and flavonoids which are considered anti-inflammatory. While the high dietary fibre content supports gut health and the growth of microbial species, potentially regulating the inhibition or production of pro-inflammatory chemokines and cytokines (Davis et al., 2020) and omega-3 PUFAs found in the diet, have been shown to regulate the immune response by inhibiting the activation of pro-inflammatory pathways and reducing cytokine expression (Giacobbe et al., 2020; Giacobbe and Mind, 2021). High-dose eicosapentaenoic acid has been shown to improve cognitive symptoms in Attention deficit hyperactivity disorder (ADHD) youth with low baseline levels (Chang et al., 2018, 2019), while research in animal models has demonstrated inflammation-induced reductions in neurogenesis can be prevented through omega-3 PUFAs intake (Borsini et al., 2017). In contrast, the Western dietary pattern, comprised of high intakes of ultra-processed foods, sodium, trans- and saturated fatty acids while being low in omega-3 PUFAs (Li et al., 2020; Okręglicka, 2015) demonstrates positive associations with pro-inflammatory biomarkers.

## 3 Clinical implications

As discussed extensively in this review, the examined studies demonstrate healthful dietary patterns such as the Mediterranean diet pattern as well as its individual constituents including vegetables and fruit, macronutrients such as fibre and healthy fats such as Omega-3, and micronutrients such as Vitamins A, C and E, have inverse associations with pro-inflammatory biomarkers. As such, modifying dietary intake to encompass these healthful elements as early as during childhood and adolescence represents a promising therapeutic strategy, potentially regulating the immune response and subsequently reducing the risk of adverse mental health disorders and associated co- and multi-morbid conditions later in life.

However, it is important to highlight that there is currently no consensus regarding the inflammatory biomarkers best used to represent chronic low-grade inflammation in children and adolescents, while biomarker measurement error such as sampling, storage and laboratory errors also cannot be excluded (Tworoger and Hankinson, 2006). Moreover, the majority of the studies in this review used a single static measurement of inflammation, however, inflammatory markers owing to their role in homeostasis and immune response are by nature not static and when measured in the fasting state are recognised as being insensitive and producing highly variable results. Several studies examined cohorts with underlying pathologies, with overweight/obesity (Karampola et al., 2019; Lazarou et al., 2010; Çağiran Yilmaz et al., 2019; Rouhani et al., 2016; Iannuzzi et al., 2009; Parillo et al., 2012; Damsgaard et al., 2013; Hajihashemi et al., 2014; Aeberli et al., 2006; Prihaningtyas et al., 2019; Machado et al., 2015; Miller et al., 2016), and type-1 diabetes (Liese et al., 2018; Sanjeevi et al., 2018; Jaacks et al., 2014) being the most studied. In overweight and obese populations excess adipose tissue has been linked to an increase in sub-chronic levels of key pro-inflammatory cytokines, mainly CRP, IL-6 and TNF-α (Reilly and Saltiel, 2017), and this may prevent or attenuate any potential therapeutic effect exerted by a healthful diet (Monteiro and Azevedo, 2010). The evident associations were also attenuated by several different factors (Calder et al., 2011; Navarro et al., 2016; Cunningham-Rundles et al., 2005). For example, gender differences were evident across the studies however, they were not specific to one particular diet, food group, macro- or micronutrient. Nor were they specific to any inflammatory biomarker. This may be in part attributable to the influence of hormones. Sex hormones affect immune function, whereby oestrogens stimulate auto-immunity and androgens exhibit protective properties (Zolin et al., 2015; da Silva et al., 2017; Whitacre, 2001; Ortona et al., 2016). These implications all need to be addressed in future research studies. Moreover, there is limited evidence examining the associations between dietary intake and low-grade inflammation in the context of neuropsychiatric disorders. Only one study included in our review examined the mechanistic pathways between dietary patterns, inflammatory markers and outcomes of depressive symptoms (Oddy et al., 2014), reporting a healthful diet was protective in these pathways. Ultimately, further interventional research is needed to establish the strength of associations between dietary intake and inflammatory biomarkers, as well as studies that examine the biological mechanisms underlying and attenuating such associations. Moreover, research studies should extend to include the examination of these associations specifically in the context of neuropsychiatric conditions and associated co- and multi-morbid conditions in children and adolescents.

## 4 Conclusion

In this review, I have discussed the current evidence that supports a potential therapeutic role for dietary intake in the amelioration of low-grade inflammation in children and adolescents. These data suggest adherence to healthful patterns and their constituents are associated with decreased levels of inflammatory biomarkers, including CRP, IL-6 and TNF- α, in children and adolescents. However, it is important to highlight the need for further research studies, that address methodological implications and factors that may confound associations, to develop a better understanding of the biological mechanisms underlying and attenuating the associations between dietary intake and low-grade inflammation in children and adolescents, specifically in the context of mental health.

## Funding

Melissa Bujtor is funded by the UK 10.13039/501100000265Medical Research Council (grants MR/N029488/1). This project has received funding from the European Union's Horizon 2020 research and innovation programme under Grant Agreement No 848158.

## Declaration of competing interest

The author declares no conflict of interest.
